# Predictability analysis of the Pound’s Brexit exchange rates based on Google Trends data

**DOI:** 10.1186/s40537-020-00337-2

**Published:** 2020-09-18

**Authors:** Amaryllis Mavragani, Konstantinos Gkillas, Konstantinos P. Tsagarakis

**Affiliations:** 1 Department of Computing Science and Mathematics, Faculty of Natural Sciences, University of Stirling, Stirling, FK9 4LA UK; 2 Department of Business Administration, University of Patras, University Campus–Rio, P.O. Box 1391, Patras, 26500 Greece; 3 Business and Environmental Technology Economics Lab, Department of Environmental Engineering, Democritus University of Thrace, Vas. Sofias 12, 67100 Xanthi, Greece

**Keywords:** Big data, Dollar, Euro, Exchange rates, Google Trends, Internet behavior, Pound sterling, Predictability analysis

## Abstract

During the last decade, the use of online search traffic data is becoming popular in examining, analyzing, and predicting human behavior, with Google Trends being a popular tool in monitoring and analyzing the users' online search patterns in several research areas, like health, medicine, politics, economics, and finance. Towards the direction of exploring the Sterling Pound’s predictability, we employ Google Trends data from the last 5 years (March 1st, 2015 to February 29th, 2020) and perform predictability analysis on the Pound’s exchange rates to Euro and Dollar. The period selected includes the 2016 UK referendum as well as the actual Brexit day (January 31st, 2020), with the analysis aiming at analyzing the Pound’s relationships with Google query data on Pound-related keywords and topics. A quantile dependence method is employed, i.e., cross-quantilograms, to test for directional predictability from Google Trends data to the Pound’s exchange rates for lags from zero to 30 (in weeks). The results indicate that statistically significant quantile dependencies exist between Google query data and the Pound’s exchange rates, which point to the direction of one of the main implications in this field, that is to examine whether the movements in one economic variable can cause reactions in other economic variables.

## Introduction

On January 22nd, 2013, David Cameron, UK’s Prime Minister at the time, stated that he would hold a referendum if he were re-elected in UK’s 2015 general elections [1]. Three years later, on February 20th, 2016, he officially announced that a referendum was to take place on June 23rd, 2016. The question that the British citizens were asked to answer to was: ‘*Should the United Kingdom remain a member of the European Union or leave the European Union?*’, with the response options being *‘Remain a member of the European Union’* and ‘*Leave the European Union’*.

Since 1973, the year that the UK became an EU member, only one referendum had been held-in 1975-where the country’s membership in the EU was put to the voting population’s deciding. The question was: ‘*Do you think that the United Kingdom should stay in the European Community (the Common Market)?*’, with about 67% of the population voting for staying in the EU [2].

David Cameron had let the Tories to freely take sides, with him being in the ‘Remain’ camp along with several other members of his cabinet, while some high-ranking members of his party were opposed to the UK staying in the EU. The Loyal Opposition, i.e. the Labour Party (though with few exceptions), along with the Liberal Democrats and the Greens, were officially in the ‘Remain’ camp. There have been suggestions that one of the reasons leading Cameron to decide to hold the referendum was to soften the Euroskeptics’ reactions within his party [3], or, in other words, the referendum calling could be viewed as ‘*a domestic political move*’ [4].

In general, UK’s voters do not feel culturally close to the EU [5], with over one-fourth of the Britons suggesting that *‘European unification has already gone too far’* [6]. How far back can UK’s euroskepticism be traced? Has it affected the UK’s view of the EU over the course of the past decades? Is EU an asset or a burden in UK’s development? These questions need to be addressed in light of the 2016 British Referendum.

Furthermore, is  Euroskeptisim only a UK’s issue or is it reflected in the previous referendums concerning membership in the EU? In general, euroskepticism seems to be high (3rd factor after ‘*perceived ethnic threat*’ and ‘*political distrust*’) in explaining the right-wing shift in voting in the EU [7]. There has been a surprisingly high number of referendums in European countries on various issues, e.g. membership, currency, constitution etc. [8], with most of them resulting in favor of the EU. However, the ‘No’ votes in the referendums have taken the upturn since the ‘90 s [8]. This increase in the ‘No’ voting could not be wholly attributed to dissatisfaction with the EU. It has been suggested that it could be a way of the citizens’ expressing their disappointment with the government [6], as a factor playing a critical role in a referendum result is how long the government has been in office [8].

Several factors had to be taken into account by British citizens when deciding on whether to ‘Remain’ in or ‘Leave’ the EU. Said factors could positively or negatively affect UK’s economy and national and international affairs, in addition to any potential consequences on the EU’s economic situation, stability, or unity. For this reason, the UK Referendum had received wide international attention both from the media [9–12] and the scientific community [5, 6, 13–16] over the months leading to the race, with both sides-pro and against Brexit- arguing over the key issues that would be of concern on the day after. Are the economic benefits associated with the EU the factors that could have made British voters overcome issues about migration and security?

Two of the main issues that affected the voters in being skeptical towards the EU were border and immigration control (52%), and welfare and benefits limitations (46%) [6]. Did the existing Schengen status provide protection against illegal immigration? The ‘Leave’ campaigners suggest that UK’s policy on migration would be better if the UK was not an EU member [6]. The rise in percentages of parties in the far right are common in recessions [17], while in the EU their main rhetoric is the anti-immigrant policies. One of the main concerns of the ‘Leave’ campaigners was the immigration levels and how they could be of concern to their security [18], as it is suggested that the UK could have the choice of not providing asylum to immigrants or other asylum seekers if it was to leave the EU [18]. Though UK is quite independent in terms of foreign policy and security [19], the ‘Remain’ supporters argued that UK could better handle terrorism and cross-border crimes from within the EU [6]. With UK having one of the two best militaries in the EU, US’ concern on the EU-NATO relations should a Brexit occur [13] is accounted for. Overall, it was  argued that in both cases-‘Remain’ or ‘Leave’-UK would continue to be able to have a separate security and defense policy [19].

As far as the economic factor is concerned, an overall calculation of any potential economic cost of a Brexit is unrealistic [3]. On the one hand, the ‘Remain’ campaigners suggested that economy, trade, and employment are positively influenced by UK’s being an EU member [6]. UK’s major trading partner is the EU, with UK’s imports at 53.1% and exports at 43.7% [20]. Trade liberalization, EU’s single market, and the increased competition have been proven valuable to UK’s economy. In the wake of a Brexit, UK would have to not only discuss the terms in trade with the EU, but with third countries as well [3].

On the other hand, the ‘Leave’ campaigners all the more suggest that it is way too costly for the UK to remain in the EU. Did the UK offer more than what was  gaining by being an EU member, or was it the case that a Brexit would be harmful for UK’s economy? UK’s contribution to the EU budget -the third after Germany and France- was estimated at 12.9% in 2015 [20], while about 33.3 billion pounds are spent every year in order for UK’s firms to comply with EU regulations [20]. When the UK exits the EU, it could change back or not proceed with applying regulations forced by the EU, though even this could be costly [3], and the freedom to not obey to EU regulation in issues like health, social issues, and climate change could be of economic benefit for the UK [16]. Though UK has witnessed significant improvement in its economy since it entered the EU, it is suggested that issues like inflation and unemployment would be better handled outside the EU [16].

Nevertheless, the possible impacts of a Brexit in the EU should not be overlooked. Could a Brexit be EU’s beginning of the end? Will the unity of the European countries remain should a security crisis were to occur? One of the key concerns is that a Brexit could harm EU’s position as a ‘*Global security actor*’ [21], or that a Brexit could lead Scotland to decide to leave Great Britain [13]. Another main concern following a Brexit is that it could lead to other countries holding referendums to leave the EU as well [13]. Will a Brexit be ‘contagious’? Let us not forget the recent example of Greece that held a referendum in 2015, that, though asking on whether or not Greeks agreed with the bailout terms proposed by the EU, it was in a sense an indirect question of whether they should remain in or leave euro and, potentially, the EU. Would Greece falsely compare the situations and conclude that a Grexit is possible? A Brexit could also weaken the argument for future EU members that entering the EU is the right choice for unity, development, and security. Other non-EU European countries, like Norway or Switzerland, would fear that they would lose their special status concerning their relations to the EU [13]. Russia could find its way to increase its influence in Eastern Europe [14], especially in light of Ukraine’s interest to enter the EU. Overall, it is possible that the balance within the EU could be overturned due to a Brexit, with any possible consequences in EU’s defense.

In any case, in the UK’s exiting the EU, the agreement that the UK negotiates should carefully take into account any potential consequences in Britain’s economy and national security. On the day of the Referendum race, having the highest turnout since the April 1992 general elections [12], about 72% of the voting population went to cast their vote. The official results, putting Leave at 51.9% and Remain at 48.1% [22], were announced the next day, unfolding a quick sequence of events, with David Cameron resigning from Prime Minister and leaving his successor, Theresa May, and the Tories to deal with a highly polarized country and a Brexit in the making.

As early as on June 24th, the market had reacted to the news of a-not yet official-Brexit, showing the forthcoming impacts of a Brexit on UK’s economy, minding that the Pound Sterling’s value-UK’s currency-reached its lowest point in 31 years that day [23]. The banks were already beginning to think of the next day, considering to even leave the UK and move to other European countries. In addition, given the rise of right-wing parties in many EU countries, EU’s stability as a whole was at stake. Following the previous year’s Greek Referendum and as the overall anti-EU rhetoric had been increasing, the fear that other countries would try to follow Britain’s example was of concern. As was evident, the vote for Brexit alone, regardless of when Article 50 would be triggered and what the outcome of the negotiations to come would be, brought along a wide public reaction in the UK.

**Fig. 1:**
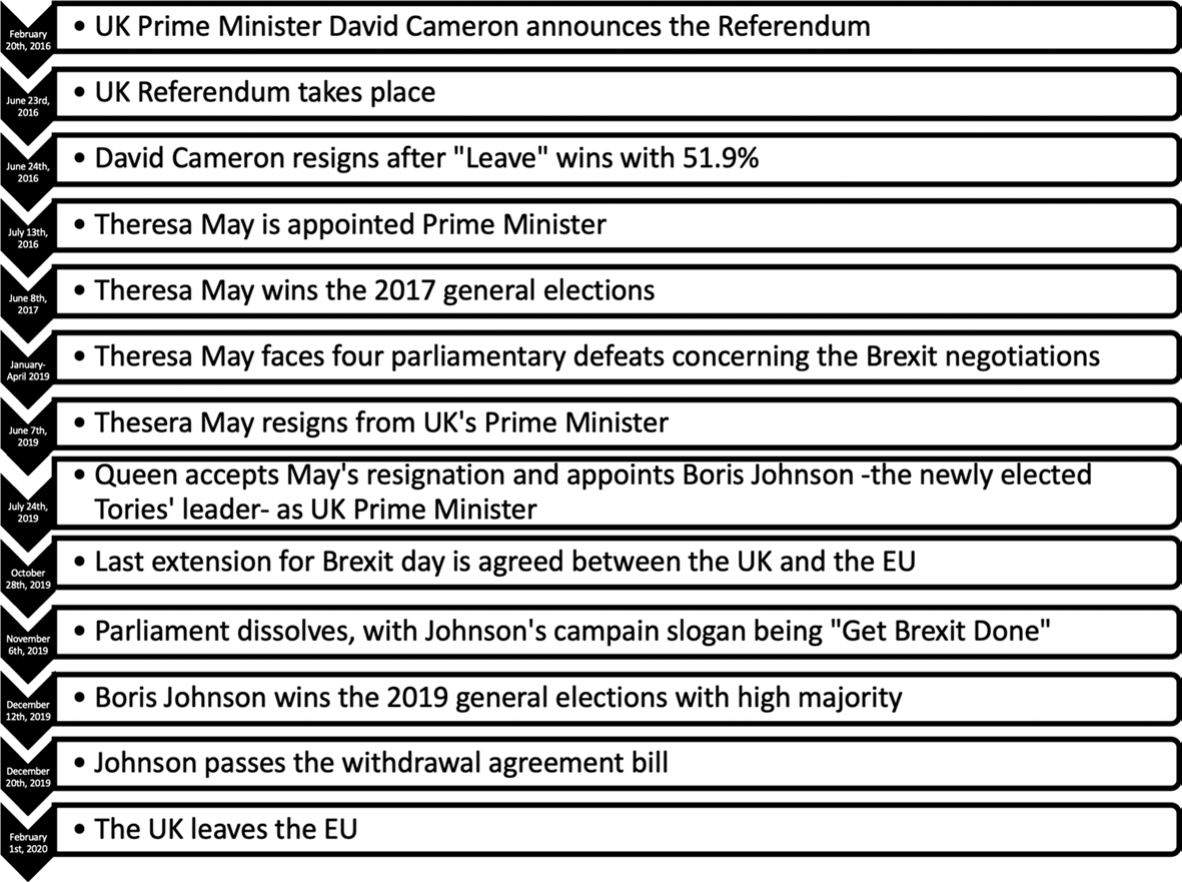
Timeline of the Brexit milestones

Following the events of June 23rd, 2016, the Tories were assigned the difficult task of implementing Brexit, with Theresa May being appointed UK’s prime minister in July, losing several parliamentary battles as to the Brexit course of action, and finally resigning in June 2019. Boris Johnson, former London Mayor and one of the most vibrant “Leave” members of the Conservative party during the 2016 UK referendum, becomes UK’s Prime Minister in July 2019, leading the country to general elections with the campaign slogan “Get Brexit Done”, and wins the Tories the highest majority since Thatcher’s win in 1987. After short negotiations with the EU and parliamentary permission to proceed with Brexit, the latter formally occurs on February 1st, 2020, where the UK is longer not a member of the EU after 47 years. Figure 1 consists of the timeline of the most important Brexit milestones since the announcement of the 2016 UK referendum.

In order to examine how the Britons, as well as the rest of world, reacted to the news of a Brexit in relation to the fear of the pound plummeting in terms of the changes in the Pound’s exchange rates after the referendum, in this study online search traffic data from Google are used, exploring the full potential of Big Data [24–26]. Such analyses have become very popular over the course of the last decade [27, 28], with the analysis of Google Trends [29] and data from online sources being a valuable tool for measuring behavioral changes [30] especially as the Internet is nowadays becoming all the more important [31], giving us the opportunity to use information that would never be accessible otherwise.

Google Trends is a tool that has been highly employed in various research fields [32] over the past decade, while it has been shown that it is significantly valuable in analyzing online interest [33]. Its accuracy and validity in forecastings [33–37], predictions [38, 39], nowcasting [40], and in examining human behavior in general has been highlighted. Economics and finance are among the main fields where online search traffic data from Google have been employed to analyze and predict behavioral variations, mainly for examining behavioral changes in stock market related issues [15, 41–47], currencies [48, 49], in general consumption [50] and sales [51–53], in general applications for advertising, marketing, and management [54, 55], or even examining the effect of taxation in the public’s view of the future [56].

In this work, we analyze the relationships between Google Trends data on Pound-related keywords and topics and its respective exchange rates with the Euro and the US Dollar over the past 5 years; a time-frame including the 2016 UK referendum as well as the actual Brexit day. Towards this direction, we employ predictability analysis on the Pound’s exchange rates, in order to further elaborate on the quantile-specific dependency by controlling various market phases and regimes between online and exchange rate data. One of the main implications is to examine whether the one variable is statistically dependent across the entire range of its conditional distribution on various levels of the other variable, which is a key issue in decision making, i.e., in economic policy and investment decisions.

The rest of the paper is structured as follows: “Data and methods” section consists of the detailed description of data collection and the methodology followed for the analysis; in “Results” section the results are presented; “Discussion” section consists of the discussion of the main findings and the limitations of this work; and “Conclusion” section consists of the concluding remarks and future research suggestions.

## Data and methods

To ensure the robustness of the results when analyzing online search traffic data, the examined terms should be carefully selected [57, 58]. For the terms to be analyzed in this study, the results in Google Trends for the search terms ‘Euro to Pound’ and ‘Pound to Euro’ (placed in quotes) significantly differed, with the term ‘Pound to Euro’ showing higher online interest; thus the latter was selected. The same was observed for ‘Pound to Dollar’ and ‘Dollar to Pound’, thus the term ‘Pound to Dollar’ was selected. The use of symbols as keywords, i.e. ‘£ to €’ or ‘£ to $’, provided significantly lower search volumes compared to ‘Pound to Euro’ and ‘Pound to Dollar’, respectively, and thus were excluded from further analysis. Note that Google Trends is not case-sensitive.

Google Trends data are normalized over the examined timeframe and are retrieved in.csv format [59]. Data from March 1st, 2015 to February 29th, 2020 are used in this study. This time-frame was selected in order to capture the interest and exchange rates variations before the referendum took place on June 23rd, 2016, and the actual Brexit on January 31st, 2020, but the period was not extended after February 2020 so as to not overlap with increased interest or lack of interest due to the COVID-19 pandemic effects.

At first, an overall presentation of the Worldwide, the UK, and the US online interest in the examined terms is provided, along with the changes in exchange rates over the examined period. Next, daily data are retrieved for the exchange rates from the Pound Sterling to Euro and from the Pound Sterling to US Dollar [60], for which the weekly averages are calculated, in order to exactly match the Google Trends’ data weekly observations.

The aim of this paper is to explore the predictability of the Pound’s exchange rates based on Google Trends data. To this direction, seven (7) different datasets from March 1st, 2015 to February 29th, 2020 are retrieved, as shown in Table 1, consisting of the description of the two dependent variables, $y_{1}$, $y_{2}$, and the independent variables,$x_{i}$, with $i = 1, 2, \ldots , 7.$ The examined pairs are: ($y_{1}$, $x_{1}$); ($y_{1}$, $x_{2}$); ($y_{1}$, $x_{6}$); ($y_{1}$, $x_{7}$); $(y_{2} ,\;x_{3} )$; $(y_{2} ,\;x_{4} )$; $(y_{2} , x_{5} )$; ($y_{2}$, $x_{6}$); and ($y_{2}$, $x_{7}$).

**Table 1:** Descriptions of the independent and dependent variables

$\varvec{y}_{1}$	Pound to Euro exchange rate
$\varvec{y}_{2}$	Pound to Dollar exchange rate
$\varvec{x}_{1}$	“Pound to Euro” (Search term), worldwide
$\varvec{x}_{2}$	“Pound to Euro” (Search term), UK
$\varvec{x}_{3}$	“Pound to Dollar” (Search term), worldwide
$\varvec{x}_{4}$	“Pound to Dollar” (Search term), UK
$\varvec{x}_{5}$	“Pound to Dollar” (Search term), USA
$\varvec{x}_{6}$	“Pound Sterling” (Topic), UK
$\varvec{x}_{7}$	“Pound Sterling” (Topic), USA

Following, in order to explore the relationship between the dependent and the independent variable in each of the examined pairs, two-unit roots tests are employed. Table 2 consists of the unit root test statistics for testing if a time series in non-stationary and possesses a unit root considering, each of them in two different cases, i.e., with and without trend: (a) The Augmented Dickey–Fuller (ADF) test [61, 62] and (b) the Phillips–Perron (PP) test [63] for the null hypothesis of a unit root that is present in a time series. The Phillips–Perron test makes a non-parametric correction to the *t* test statistic. The test is robust with respect to unspecified autocorrelation and heteroscedasticity in the disturbance process of the test equation. The Phillips–Perron test performs less well in finite samples than the Augmented Dickey–Fuller test. The critical values at 5% and 10% significance levels as well as the p-values are also provided [in brackets]. The * and ** lead to the rejection of the null hypothesis that a time series has a unit root at 10% and 5% levels, respectively (Table 2).

**Table 2:** Unit root test statistics for the examined time series

	$\varvec{y}_{1}$	$\varvec{y}_{2}$	$\varvec{x}_{1}$	$\varvec{x}_{2}$	$\varvec{x}_{3}$	$\varvec{x}_{4}$	$\varvec{x}_{5}$	$\varvec{x}_{6}$	$\varvec{x}_{7}$
ADF test									
Test statistic	− 1.10**	− 12.10**	− 6.90**	− 6.87**	− 7.34**	− 8.24**	− 7.29**	− 7.06**	− 8.29**
Critical value	− 2.872	− 2.872	− 2.872	− 2.872	− 2.872	− 2.872	− 2.872	− 2.872	− 2.872
P-value	[0.000]	[0.000]	[0.000]	[0.000]	[0.000]	[0.000]	[0.000]	[0.000]	[0.000]
ADF test (including trend)									
Test statistic	− 11.79**	− 12.09**	− 6.94**	− 6.91**	− 7.33**	− 8.30**	− 7.36**	− 7.05**	− 8.45**
Critical value	− 3.427	− 3.427	− 3.427	− 3.427	− 3.427	− 3.427	− 3.427	− 3.427	− 3.427
P-value	[0.000]	[0.000]	[0.001]	[0.000]	[0.000]	[0.000]	[0.000]	[0.000]	[0.000]
PP test									
Test statistic	− 12.48**	− 13.08**	− 6.85**	− 6.87**	− 7.33**	− 8.24**	− 7.29**	− 7.15**	− 8.22**
Critical value	− 2.872	− 2.872	− 2.872	− 2.872	− 2.872	− 2.872	− 2.872	− 2.872	− 2.872
P-value	[0.000]	[0.000]	[0.000]	[0.000]	[0.000]	[0.000]	[0.000]	[0.000]	[0.000]
PP test (including trend)									
Test statistic	− 12.72**	− 13.07**	− 6.90**	− 6.91**	-7.32**	− 8.30**	− 7.36**	− 7.14**	− 8.38**
Critical value	− 3.427	− 3.427	− 3.427	− 3.427	− 3.427	− 3.427	− 3.427	− 3.427	− 3.427
P-value	[0.000]	[0.000]	[0.000]	[0.000]	[0.000]	[0.000]	[0.000]	[0.000]	[0.000]

Following, a quantile dependence method is employed, i.e., cross-quantilograms to test for directional predictability from Google Trends data to the Pound’s exchange rates. The latter is proposed by [64] and allows us to detect and measure directional predictability of a time series at various lags and quantiles. The advantage of this technique is that it estimates the lead-lag correlations between different time series contemporarily, at different lags and quantiles. In particular, let us consider two time-series that are defined as follows: $\left\{ {x_{t} , t \in Z} \right\}$ and $\left\{ {y_{t} , t \in Z} \right\}$ and that are strictly stationary. With regard to our study, if variable $x_{t}$ is the Google Trends data, and variable $y_{t}$ is the Pound’s exchange rates, the sample cross-quantilogram for positive values of $k$ is given by:

1 $$\hat{\rho }_{\alpha } \left( k \right) = \frac{{\mathop \sum \nolimits_{t = k + 1}^{T} \varPsi_{{\alpha_{1} }} \left( {y_{t} - \hat{q}_{x,t} \left( {\alpha_{1} } \right)} \right) \cdot \varPsi_{{\alpha_{2} }} \left( {x_{t - k} - \hat{q}_{y,t - k} \left( {\alpha_{2} } \right)} \right)}}{{\sqrt {\mathop \sum \nolimits_{t = k + 1}^{\rm T} \varPsi_{{\alpha_{1} }}^{2} \left( {y_{t} - \hat{q}_{x,t} \left( {\alpha_{1} } \right)} \right)} \cdot \sqrt {\mathop \sum \nolimits_{t = k + 1}^{\rm T} \varPsi_{{\alpha_{2} }}^{2} \left( {x_{t - k} - \hat{q}_{y,t - k} \left( {\alpha_{2} } \right)} \right)} }}$$

where the $\hat{q}_{j,t} \left( {\upalpha_{j} } \right)$, $j = x, y$ is the unconditional sample of the $x,y$ series, with $\hat{\rho }_{\alpha } \left( k \right) \in \left[ { - 1,1} \right]$. What is described in Eq. (1), i.e., the cross quantilogram-given a set of quantiles $k = 0, \pm 1, \pm 2 \ldots ,{ - },$ is in sense a calculation of the dependency as far as the deviation’s direction for said quantiles is concerned, which, in turn, quantifies the one variable’s predictability based on the other. Considering the range of values that $\hat{\rho }_{\alpha } \left( k \right)$ can take, total predictability is indicated if $\hat{\rho }_{\alpha } \left( k \right) = \pm 1$, while no predictability is indicated when it becomes zero.

## Results

**Fig. 2:**
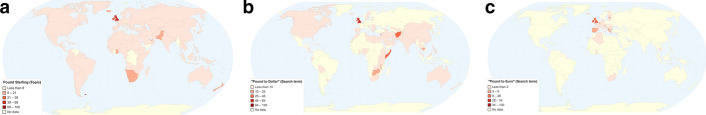
Worldwide online interest in the **a** Pound Sterling (Topic), **b** “Pound to Dollar” (Search Term), and **c** in the “Pound to Euro” (Search Term) (March 2015–February 2020)

**Fig. 3:**
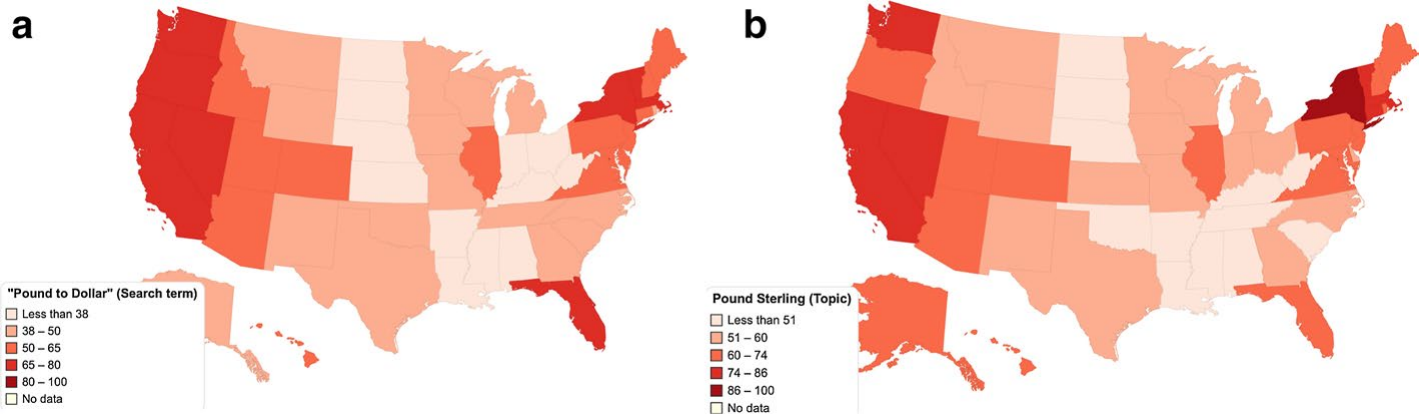
US online interest in the **a** “Pound to Dollar” (Search Term) and **b** in the Pound Sterling (Topic) (March 2015–February 2020)

Figures 2 and 3 depict the heat maps for the online interest in keywords and topics related to the Pound, Worldwide and in the US, respectively. Heat maps for the UK were not included, as for all UK countries the interest is very high, and these maps would not visually add to the results.

For the “Pound Sterling (Topic)” Worldwide, it is evident that the interest is high, especially, as expected, in the UK and Ireland. As for the examined search terms, “Pound to Dollar” exhibits significantly higher interest in terms of number of countries than “Pound to Euro”. Note that both currencies, i.e., US Dollar and Euro, are among the strongest currencies in the world.

**Fig. 4:**
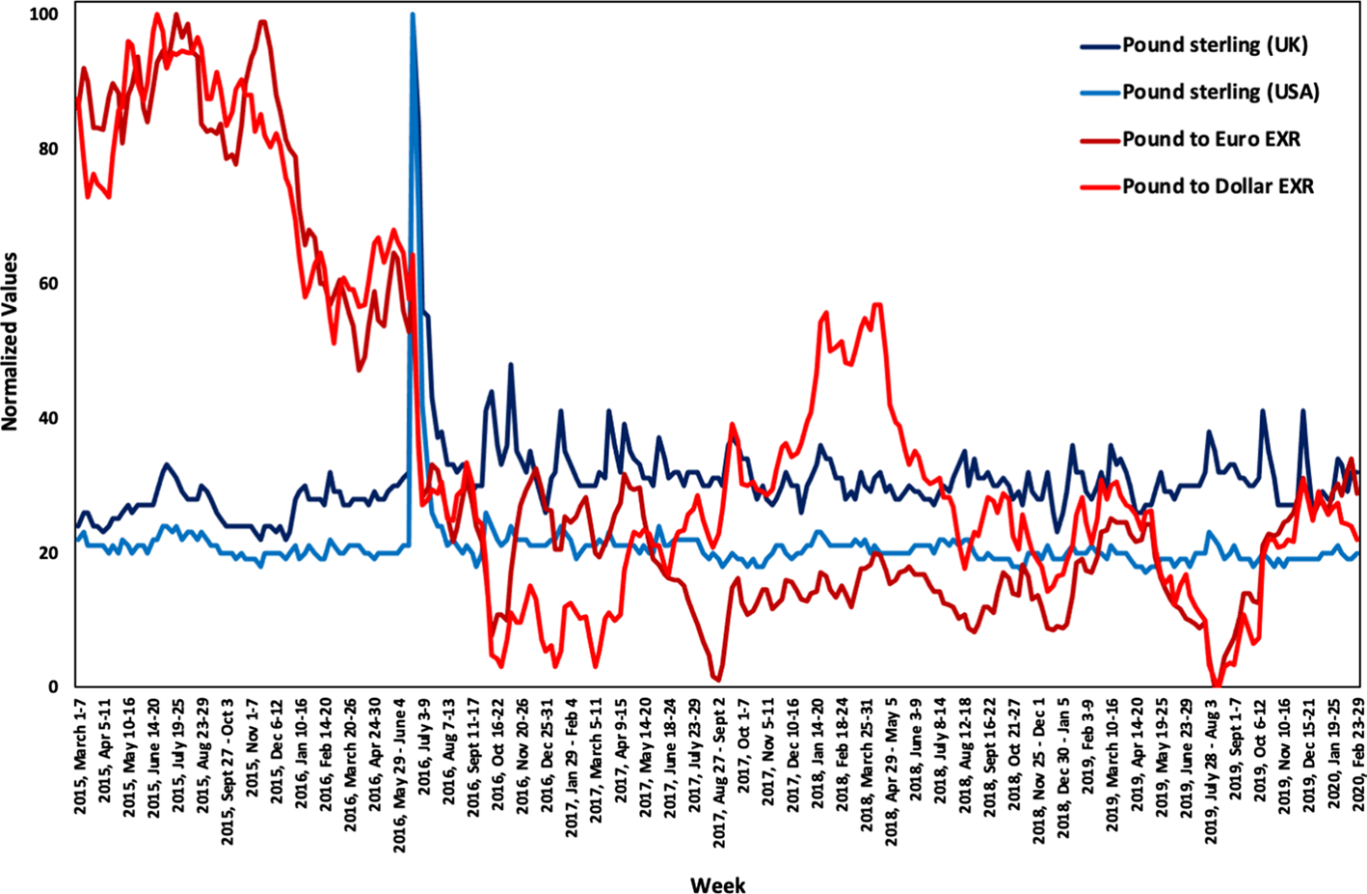
Exchange rates and online interest for the Pound in the UK and USA

Following, Fig. 4 depicts the changes in the online interest in the examined terms and topic, as well as the variations in the exchange rates (EXR) of Pound to Euro and Dollar. The exchange rates for each currency were normalized for graph consistency reasons following the formula $x^{\prime} = \frac{{x - x_{min} }}{{x_{max} - x_{min} }}100$.

As is evident, starting in 2015, the exchange rates for Pound to Euro and Dollar are the highest over the examined period, and take a sudden drop the week after the 2016 UK referendum. The exhibit a rise over the first half of 2018 and reach their overall low in August 2019. During the weeks when Brexit actually occurred more than 3.5 years later, the exchange rates reached the 2016 exchange rates again. As per the online interest, a spike during the referendum is observed, i.e., the exact same week as the sudden drop in exchange rates. During the rest of the examined period, the interest is relatively stable, and no extreme spikes are observed.

**Fig. 5:**
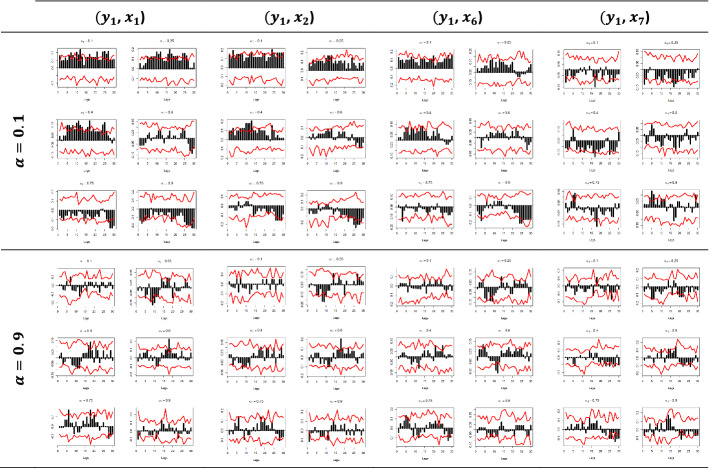
Quantile dependencies between $$y_{1}$$ and $$x_{1} ; x_{2}$$; $$x_{6} ;$$$$x_{7}$$, for $$a_{2} = 0.1$$, $$a_{2} = 0.9$$

**Fig. 6:**
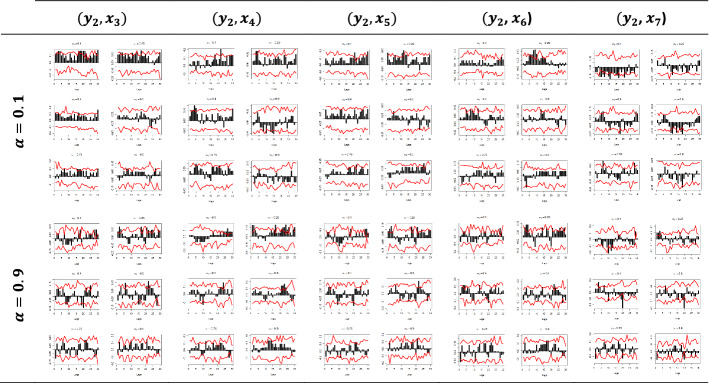
Quantile dependencies between $y_{2}$ and $x_{3} ; x_{4}$; $x_{5} ; x_{6}$; $x_{7}$, for $a_{2} = 0.1$, $a_{2} = 0.9$

Figures 5 and 6 depict the sample cross-quantilograms $\hat{\rho }_{\alpha } \left( k \right)$ between all examined pairs for the dependent variables $y_{1}$ and $y_{2}$, respectively. Said figures present the directional predictability of the Pound’s exchange rates based on the respective Google Trends time series for the low and high values of the $x_{i}$, i.e., for quantiles $a_{2} = 0.1$-indicating the smallest 10%—and $a_{2} = 0.9$-indicating the largest 10%. Note that $a_{2} = 0.4$ and $a_{2} = 0.6$ refer to the median values of the respective $x_{i}$.

**Fig. 7:**
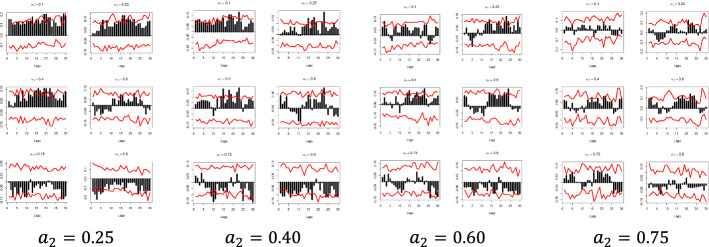
Pound to Euro exchange rate—“Pound to Euro” (Search term), Worldwide ($y_{1}$, $x_{1}$)

**Fig. 8:**
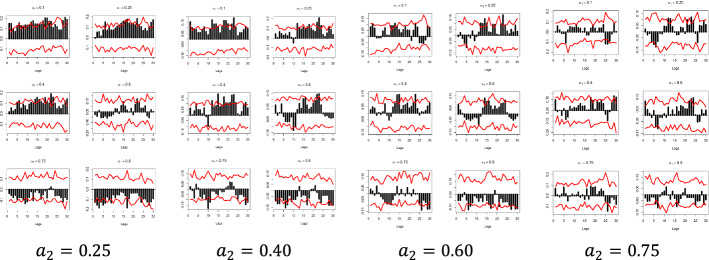
Pound to Euro exchange rate—“Pound to Euro” (Search term), UK ($y_{1}$, $x_{2}$)

**Fig. 9:**
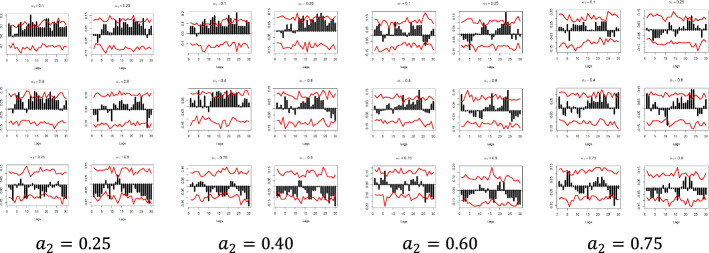
Pound to Euro Exchange rate—“Pound Sterling” (Topic), UK ($y_{1}$, $x_{6}$)

**Fig. 10:**
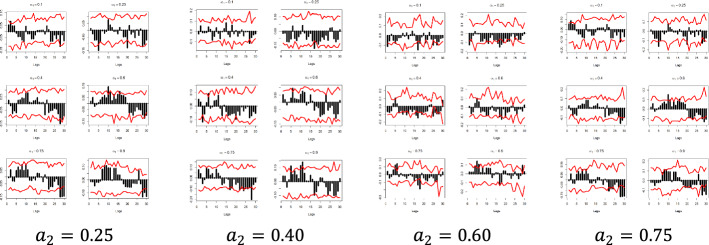
Pound to Euro exchange rate—“Pound Sterling” (Topic), United States ($y_{1}$, $x_{7}$)

**Fig. 11:**
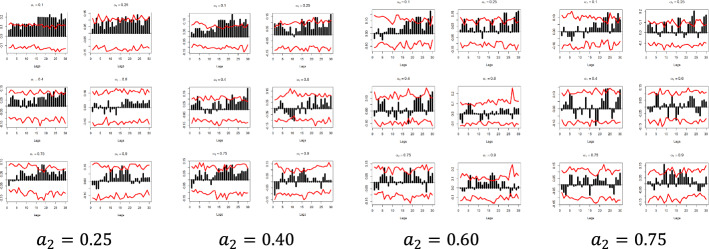
Pound to Dollar exchange rate—“Pound to Dollar” (Search term), Worldwide $( {y_{2} , x_{3} } )$

**Fig. 12:**
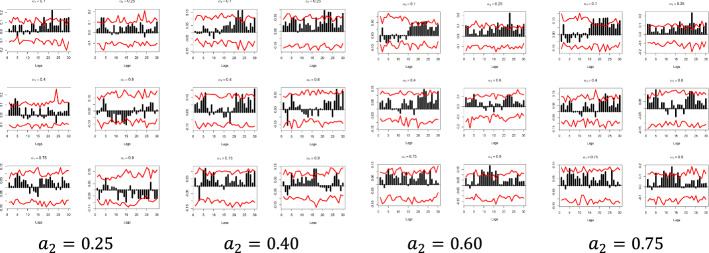
Pound to Dollar exchange rate—“Pound to Dollar” (Search term), UK $( {y_{2} , x_{4} } )$

**Fig. 13:**
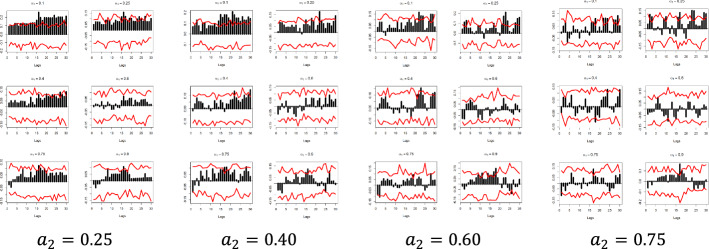
Pound to Dollar exchange rate—“Pound to Dollar” (Search term), USA $( {y_{2} , x_{5} } )$

**Fig. 14:**
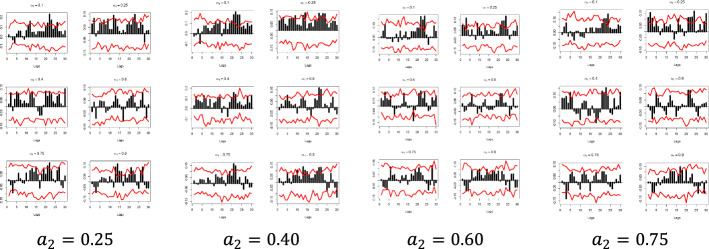
Pound to Dollar exchange rate—“Pound Sterling” (Topic), UK ($y_{2}$, $x_{6}$)

**Fig. 15:**
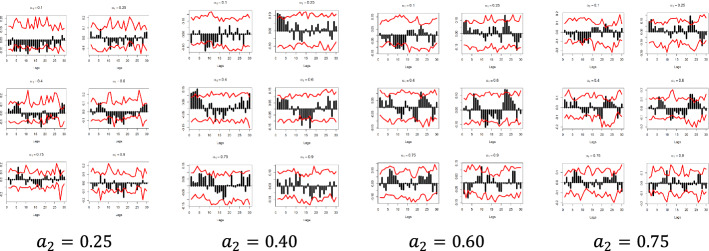
Pound to Euro exchange rate—“Pound Sterling” (Topic), USA ($y_{2}$, $x_{7}$)

The x-axis represents the lag between the two variables in weeks, the y-axis (i.e., the black bars) represents the strength of the quantile dependency between the respective variables, while the red line shows the 95% confidence interval. In this section, focus is given to the high ($$a_{2} = 0.9$$) and low ($$a_{2} = 0.1)$$ levels of the $$x_{i}$$ quantiles, but the rest of the $$x_{i}$$ quantiles ($$a_{2} = 0.25$$, $$a_{2} = 0.40$$, $$a_{2} = 0.60$$, and $$a_{2} = 0.75$$) and for all examined pairs can be found in Figs. 7, 8, 9, 10, 11, 12, 13, 14, 15 in “Appendix”.

As depicted in Figs. 5 and 6 and considering the example of $y_{1}$ at the respective $x_{i}$’s quantiles $a_{2} = 0.1$, i.e., for the lowest levels of the respective Google Trends time series, we observe that there is a statistically significant dependency with all of the $x_{i}$ variables in all quantiles and in at least one lag.

Nevertheless, the results show that the quantile dependency between the Euro exchange rates and Google Trends data is statistically significant, as shown, for example, in the ($y_{1}$, $x_{1}$) pair, for $a_{1} = 0.1$, and for $a_{2} = 0.1$, where all dependencies are positive and almost all between a 5- and a 13-week lag are statistically significant, with the dependency peaking at week 12.

The latter can be explained as follows: when Google Trends data take low values, it is possible that low values for the exchange rates will occur as well, further indicating that when the interest is low, the market can create negative returns.

Tables 3 and 4 consist of a presentation of the statistical significance of all lags for all examined ($y_{1}$, $x_{i}$) with $i = 1, \;2, \;6,\; 7,$ and ($y_{2}$, $x_{i}$) with $i = 3,\; 4, \;5,\; 6, \;7$ pairs, for quantiles $a_{2} = 0.1$ and $a_{2} = 0.9$, respectively. Note that ‘+’ denotes a statistically significant quantile dependency, while the ‘−’ denotes a not statistically significant dependency; the signs do not indicate whether or not a dependency is positive or negative. Also, the $\alpha_{i}$ in the columns refer to the $x_{i}$ while the $\alpha_{i}$ in the lines refer to the $y_{i}$ quantiles.

**Table 3:**
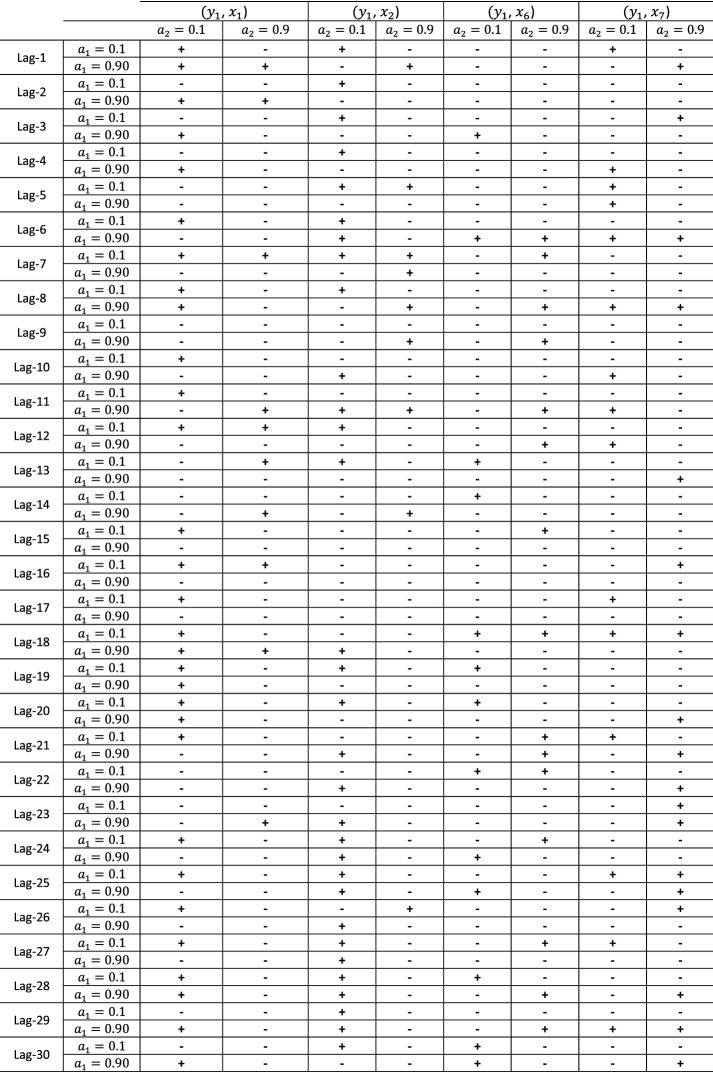
Statistical significance for all examined ($y_{1}$, $x_{i}$) with $i = 1, 2, 6, 7,$ pairs

**Table 4:**
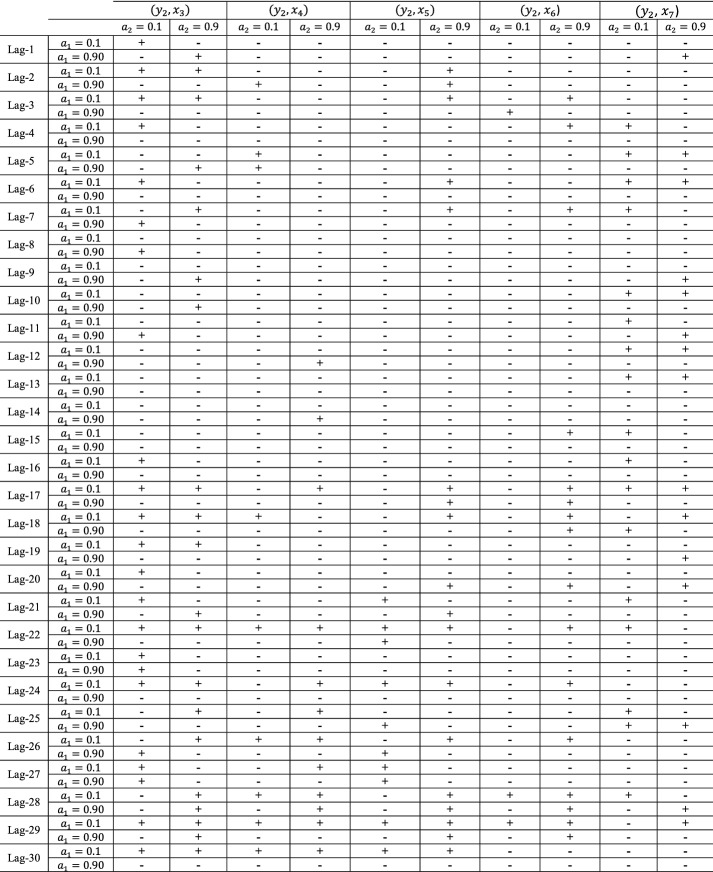
Statistical significance for all examined ($y_{1}$, $x_{i}$) with $i = 1, 2, 6, 7,$ pairs

## Discussion

Over the past decade, with the high integration of Big Data Analytics in academic research, the analysis, forecasting, and prediction using Google Trends time series have been shown to be of value in several research fields. In this paper, we examined the relationships between search queries in Google and exchange rates for the Pound Sterling to Euro and US Dollar following the 2016 UK Referendum, asking the Britons on whether or not they wished to remain in or leave the European Union. Given the wide international attention that this Referendum received, along with the potential consequences that a Brexit would have on both the British and the EU economy, a unique opportunity was presented in order to quantify and analyze the UK and Worldwide online behavior towards the Pound Sterling.

Both the UK and Worldwide online interest peaked on the Referendum week, also evident by the wide international attention that this particular race received. Supporting previous work on the subject in the field of economics and finance that have suggested that Google data have exhibited good potential in finance [42, 65, 66], our results, showing this strong dependency between the variables, indicate that the future forecasting of the behavioral variations of the Pound’s exchange rates is possible, subject to careful analysis.

The latter is also supported by the empirical relationships between online search traffic data and human behavior that have been shown to exist [27, 28, 33, 38, 67]. Despite this, we cannot argue that a direct link between a query and a change in exchange rates exists. The public’s reaction to finance-related issues, as in the 2008 recession, has been shown to be better monitored by Google data, as the lag, if any, is considerably lower than with other approaches [68].

Towards the direction of examining the predictability of Pound’s exchange rates with Euro and US Dollar using Google Trends data, the results of this study indicate that changes in exchange rates are depicted and related with online searches with a lag, with the latter depending on the which independent variables are examined. Statistically significant dependencies between Google queries and the Pound’s exchange rates exist, and these findings are supportive of previous work on the subject, where Google Trends’ data have been shown to be related to trading activity [44], and assisting in measuring the uncertainty about the state of the economy [46], in predicting stock returns [43] and in forecasting volatility [47].

This study has some limitations. At first, data from only the last 5 years were considered, and only on the Pound’s exchange rates with two other currencies. In addition, only queries from Google were retrieved and not from other search engines; however, Google is used by the vast majority of internet users. Finally, only certain keywords’ and topics’ interest was examined.

Based on the results of this study that support previous work on the subject, it is evident that online search queries from Google can be very valuable in examining the users' behavioral variations in topics related to the fields of economics and finance. Google Trends data depict behavioral changes in general and variations attributed to an event in particular and have shown great potential in not only analyzing human behavior, but in forecastings, predictions, and nowcastings as well. Google Trends strongest feature is that it provides us with the revealed and not the stated users’ preferences [39, 69], thus, though the sample is unknown, it reveals information that could not have been accessed otherwise.

Current analysis produces quantile-specific results controlling various market phases and volatility regimes. In other words, the advantage of this method is that it allows us to estimate lead-lag correlations between different time series contemporarily, at different lags and quantiles, or, to put it differently, whether or not specific quantiles values of the one variable can contemporality occur together with the values of a specific quantile of the other variable, and thus, we are able to avoid spurious results that standard casual models do not permit us to take into account. More importantly, we report a lack of predictability of crisis risk (Brexit) on future market returns employing predictive linear regressions. Indeed, our study highlights not only the importance of using statistical tools beyond the conditional mean, but also that the link between trends and exchange markets may exist in higher moments.

Our statistical analysis is based on quantiles specific results for the Sterling Pound and hence in the process of capturing various phases (sizes) of Sterling pound movements in a trading day. This is the reason why our study has various implications for investors and policymakers. One of the main targets of this study is to understand the nature of large price movements in the exchange markets and whether or not trends can have a predictive power on it. Primarily, investors make their investment decisions associated with risk management and designing an appropriate asset allocation strategy. However, their decision behavior can be changed by their subjective perception on the arrival of new information due to different expectations. Finally, taking into account that policymakers have to make decisions during periods of turbulence in exchange markets, it is economically vital to progress in a better econometric/statistical understanding when extreme markets movements happened in accordance with their real determinants [70].

## Conclusions

Given the wide national and international attention that Brexit has received over the past years, it is important to explore the relationships of the Sterling Pound’s exchange rates with several dependent variables. To this direction, this paper at first examines the online behavioral variations based on Google Trends data on selected keywords and topics, and aims at exploring the Pound to Euro and Pound to Dollar exchange rates’ predictability following the 2016 UK Referendum where the Britons were asked to vote whether or not they wished for the UK to remain in the EU. Employing the quantile dependence method of cross-quantilograms for data from March 2015 to February 2020 and for lags from zero to 30 (in weeks), we found that statistically significant quantile dependencies exist between the Pound’s exchange rates and the respective Google query keyword and topic data.

The observed dependencies exhibit very promising results in the predictability of the Sterling Pound and highlight the importance of using web-based sources in order to address the information excess in the Big Data Era. Such predictability analyses can become a key policy factor in currency monitoring, as the long-term relationship of the Pound’s exchange rates with Google Trends data suggests that the exchange rates could be nowcasted using online search traffic data in the future.

Future approaches should explore the Pound’s predictability using other online sources, like Twitter or other popular Social Media, and also focus on employing a combination of variables, in order to take full advantage of what the internet has to offer. Similar methodologies should explore nowcasting approaches, as data from web-based sources can provide us with real-time assessment. Though the present analysis only focuses on the Pound to Euro and the Pound to Dollar exchange rates, future research can include more monetary variables, countries, currencies, and keyword combinations, in order to better explore and take advantage of the possibilities rising from the increasing use of the Internet.

## Data Availability

All data used in this study are publicly available and accessible in the cited sources.

## Appendix

Figures 7, 8, 9, 10, 11, 12, 13, 14, 15 depict the $x_{i}$ quantiles for $a_{2} = 0.25$, $a_{2} = 0.40$, $a_{2} = 0.60$, and $a_{2} = 0.75,$ for ($y_{1}$, $x_{1}$); ($y_{1}$, $x_{2}$); ($y_{1}$, $x_{6}$); ($y_{1}$, $x_{7}$); $(y_{2} ,\;x_{3} )$; $(y_{2} , x_{4} )$; $(y_{2} ,\;x_{5} )$; ($y_{2}$, $x_{6}$); and $(y_{2}$, $x_{7}$), respectively.
